# The Role of Digital School-Home Communication in Teacher Well-Being

**DOI:** 10.3389/fpsyg.2019.02257

**Published:** 2019-11-14

**Authors:** Anne-Mari Kuusimäki, Lotta Uusitalo-Malmivaara, Kirsi Tirri

**Affiliations:** ^1^Faculty of Educational Sciences, University of Helsinki, Helsinki, Finland; ^2^Department of Education, Helsinki Collegium for Advanced Studies, University of Helsinki, Helsinki, Finland

**Keywords:** parent–teacher partnership, digital communication, teacher well-being, teacher education, Finnish schools

## Abstract

Well-functioning communication is crucial in all work communities. A respectful and trusty partnership between parents and teachers in schools is essential not only for pupils but also for the well-being of the whole organization. Communication is at the heart of such a partnership. Although most parent-teacher communication nowadays takes place on digital platforms, not much is known about the specific role of digital communication (DC) in building parent–teacher partnerships. In an attempt to find out we asked 400 Finnish parents and 80 teachers about their experiences of communicating digitally and the kind of matters they discussed, and how they thought feedback on pupils should be expressed. The data was content-analyzed. Three categories related to DC content were identified: study-related matters, behavioral issues, and sensitive issues. Parental and teachers’ expectations of how pupil feedback should be expressed also fell into three categories: a good balance of encouraging and corrective feedback, more encouraging feedback, and a constant emphasis on the child’s weaknesses. These results have research and practical implications for teacher well-being.

## Introduction

The aim in this paper is to find out how digital communication (DC) can benefit teacher well-being and parent–teacher partnerships in Finnish schools. Parents (*N* = 400) and teachers (*N* = 80) from Finland responded to open questions related to their experiences of DC as part of a larger study. A new, specially designed 14-item Digital Communication Scale (DCS) was used in a recent study (Kuusimäki et al., 2019) to elicit the opinions of Finnish parents (*N* = 1123) and teachers (*N* = 118) on DC in urban and rural areas. According to the results, the parents and teachers were satisfied with the communication, which they perceived as supporting the parent–teacher partnership and providing valuable information on the development of pupils and on issues related to their schooling. However, the parents stated that the feedback they received about their children was less encouraging than the teachers thought it was. On the other hand, the teachers experienced more ambiguity in DC than the parents did. In general, rural parents and teachers were more positive about the effect of DC on their collaboration than their urban peers (Kuusimäki et al., 2019). Our aim in this article is to build on the results of that study and explore further the aspects of parent-teacher communication that can enhance teacher well-being. Our specific emphasis is on the nature of the DC that can support the well-being of teachers in Finnish schools.

Teacher stress and burnout are rather well-studied research areas (Maslach et al., 1996; Stoeber and Rennert, 2008; Benevene and Fiorilli, 2015; Benevene et al., 2018). Work engagement and professional dedication are sources of life satisfaction, but they may also be burdening (Hakanen et al., 2018). According to the European Working Conditions Survey (2015), employees in human-service jobs such as education report higher work engagement than those in several other job areas. Demands on schools and the growing heterogeneity of pupils and their homes seem to increase the challenges in the work of teachers (Chang, 2009; Skaalvik and Skaalvik, 2011). In a survey conducted by The Trade Union of Education in Finland (OAJ, 2017), 59% of teachers reported too heavy a workload, and 43% suffered from stress quite or very often. Starting from the beginning of the 2010s, well-being instead of ill-being has been the focus of a plentitude of studies (e.g., De Pablos-Pons et al., 2012). Teacher well-being is positively related to a good working community (Konu and Rimpelä, 2002), a fair share of tasks, support from the principal, and smooth collaboration with parents (Skaalvik and Skaalvik, 2011).

Teacher wellbeing is a critical issue in Finnish society, particularly given the decline in applications for teacher education. For the past 40 years Finnish teacher education has attracted gifted students whose first choice was the teaching profession (Tirri, 2014), a trend that is unique to Finland and could be attributed to historical and societal factors. The profession has thus far been more respected in Finland than in other countries. The current decline in applications may reflect a shift in the respect that the teaching profession attracts, or the circumstances in schools with diverse families and increasing demands for teacher competence in DC. Furthermore, the implementation of the new Finnish national curriculum (NBE, 2014), which among other developmental goals requires close school-home collaboration, has resulted in the feeling among many teachers that they do not have enough resources to manage well in their jobs (OAJ, 2017).

Joyce Epstein’s seminal work (Epstein et al., 2009) on school–home collaboration underlines the shared responsibility between parents, teachers, and communities in taking care of pupils’ holistic development. She established the importance of frequent interaction between schools and homes in *Theory of Overlapping Spheres of Influence* (Epstein, 1995). According to Epstein (2011), a successful parent–teacher partnership has several positive effects on the well-being and academic achievements of pupils (see also Samdal and Torsheim, 2012). Parents play an essential role in a school community, and a well-functioning partnership between parents and teachers constitutes the basis of the child’s social learning environment (see also, Samdal, 1998; Konu and Rimpelä, 2002). Moreover, the more contact with the school and involvement in their children’s studies parents have, the more likely are the children to do their homework, succeed in tests and develop positive attitudes toward school (Bauch, 1998; Freytag, 2001). Thus, parents’ strong support to their child’s studies can have a major impact on a teacher’s work and well-being. Smooth communication between parents and teachers is a prerequisite for a fruitful companionship.

### Digital Communication

Ways of maintaining contact between schools and homes include parental evenings, face-to-face meetings, phone calls, printed letters, e-mails, text messages, and school web pages. The most commonly used communication channel in Finland nowadays is a digital platform through which the great majority of information is delivered. On the platform, teachers can inform parents about the timetables and events in the school, provide shorter or longer feedback on pupils’ progress and discuss diverse topics with parents (Kuusimäki et al., 2019). Overall, digital platforms enable exchanging information about all running issues but also about more personal matters such as pupil progress, possible conflicts in school or learning difficulties (Carr et al., 2015; Palts and Kalmus, 2015). At its best, parent–teacher partnership is built with respectful two-way communication with frequent, trusty interaction strengthening the idea of striving toward common goals (Epstein et al., 2009). DC ensures rapid messaging, but it simultaneously has its challenges. As Epstein et al. (2009) found, essential elements of efficient parent–teacher communication include *clarity, readability, frequency, quality, effectivity, and informativity*. We also identified these aspects of effective DC in our previous study (Kuusimäki et al., 2019). Juniu (2009) adds four aspects that should be considered in an optimal parent-teacher collaboration: (a) positive communication including messages of encouragement from teachers to parents, (b) personalized communication, letters or feedback on the pupils’ work, (c) proactive communication, and (d) partnership and collaboration between parent and teacher (Juniu, 2009). Below we explore these aspects further with regard to Finnish parents and teachers.

### Advantages and Disadvantages of DC for Teacher Well-Being

Digital communication has many advantages over traditional forms of communication, facilitating timely online information exchange and immediate feedback between parents and teachers, for example. It also makes it easier to intervene in pupils’ learning and behavioral problems at an early stage, and thereby to improve study outcomes (Carr et al., 2015). Complementing the positive outcomes among pupils, effective and frequent two-way communication supports positive partnership among parents and teachers in that both receive information about what is going on at school and at home (Chu, 2018). DC could also encourage parents living separately to participate in school activities, both being able to access the digital platform (Palts and Kalmus, 2015), and it allows parents and teachers to communicate at times that suit both parties. Other advantages include easy information storage and being able to communicate with the entire school community simultaneously (Ramirez, 2001; Palts and Kalmus, 2015), as well as enhancing work satisfaction, motivation, and the pedagogical atmosphere among teachers by allowing continuous parental support of their daily work with pupils (Scheopner, 2010; De Pablos-Pons et al., 2012; Oostdam and Hooge, 2013).

However, communication between parents and teachers is not always seamless. Given the limited expertise in DC among teachers, the lack of time during the school day and the chances of being misunderstood, problems may well arise. DC lacks exact rules, it is time-consuming, and it follows teachers from school to home thereby blurring boundaries between work and leisure (Agger, 2011; Palts and Kalmus, 2015). The idea of always being available and within reach may seriously affect teacher management of working time, work-related stress, and consequently teacher well-being (Skaalvik and Skaalvik, 2011). Moreover, experiences of not being trusted or of being criticized by parents may cause anxiety and feelings of inadequacy among teachers (Skaalvik and Skaalvik, 2011). Consequently, good communication skills are needed to prevent unnecessary conflict with parents and to build a positive partnership, mutual respect, and trust among all families (Epstein, 2013). Teachers require pedagogical sensitivity when pondering upon whether DC is the appropriate way to contact parents, especially when delicate matters need to be discussed. It should also be remembered that parental opportunities and competences with regard to the use of technology depend on age, education, income, and cultural background. Poor language skills may be a barrier, resulting in unequal communication opportunities. Schools should ensure that all parents understand the information they receive (Carr et al., 2015). DC also demands emotional and writing competence, given that misunderstandings occur more easily than in face-to-face communication (Palts and Kalmus, 2015).

In addition to reflecting on the advantages and disadvantages of DC, one needs to consider the content and the quantity of messages. Parents require frequent information regarding their children’s overall progress with their studies, namely their grades, attendance record and homework (Freytag, 2001; Fusco, 2004; Weinstein, 2005), thereby enabling them to provide more active support of schoolwork. Another significant element of DC concerns the socio-emotional development of pupils. Parents should have an open and proactive dialog with teachers about their children’s behavior and about issues affecting well-being such as unhappiness or problems making friends (Juniu, 2009). Without open dialog, conflicts may arise. Common agreement about the content and quality of DC should be negotiated in schools to ensure a congruent policy on informing parents.

Parents need (NBE, 2014) and want (Kuusimäki et al., 2019) supportive feedback on their children’s development and studies. However, it seems as if feedback is unevenly given and gender-dependent. In Finland, Oinas et al. (2017) analyzed 211,003 digital teacher-to-parent quick-markings evidencing that boys more than girls get negative feedback and the majority of pupils get encouragement only occasionally. According to Oinas et al. (2017), teachers’ feedback on pupil performance should be realistic and concrete so that parents know how to support their children in learning. Additionally, digital feedback should be sensitive, building common understanding, creating a positive learning environment, and increase school adjustment (Reddy et al., 2003). Hence, demands for a thriving DC are high and they can easily add stress in teachers. In particular, being criticized by parents online may severely affect teacher well-being.

The aim of the current study is to provide more detailed information on the views of parents and teachers on DC and how they contribute to teacher well-being. We build on the results of our previous study on digital parent-teacher communication (Kuusimäki et al., 2019) and explore further the aspects of contents of messages and form of feedback they deliver.

Specifically, we aim to find out:

(1) what kind of contents parents and teachers wish to communicate digitally, and
(2) how pupil feedback should be expressed in DC.

## Materials and Methods

### Ethics Statement

In our research, we followed the guidelines of the Finnish Advisory Board on Research Integrity (2009). This means that we respected voluntariness, anonymity, and confidentiality related to our participants and informed them in advance of the details of our study and asked for inform consents from them. According to Kvale (1996), qualitative research needs to include the following aspects to acknowledge research ethics: inform consent, confidentiality, and knowledge of the consequences of the study. First, an invitation to participate in the study was sent to two selected municipal education administrators in April 2016 as part of the larger study. We asked the permission from administrators to approach the schools selected to our study. After that, the administrators sent an informative letter to school principals and asked their consent to approach the teachers and parents of their schools. The administrators were then asked to send principals the web link to the questionnaire and principals were asked to forward the link to the teachers and the parents of the children in their schools. The administrators received two reminders about the study and the online questionnaire was open 3 weeks in total. The link was sent through the digital platform that schools are using to communicate with parents. Parents and teachers have private passwords to enter the digital platform in order to ensure the privacy of communication and in this case answering the questionnaire. Participants were informed about the voluntariness and preserving the privacy of each participant.

Our study did not deal with animals or any vulnerable groups, nor did it involve risks for participant well-being, or use of biomedical devices, or invasive investigation tools. Our study did not need ethics approval, according to our national regulations as well as to the Ethical board of the University of Helsinki.

### Participants and Procedure

The participants of the current study were 400 parents and 80 teachers from Finland. These respondents answered to open qualitative questions related to their experiences of DC as part of a larger quantitative study. Cities selected to this study were samples of rural and urban areas, giving a relatively diverse overview of one of the largest and one of the medium-sized municipalities in Finland. Participants were parents and teachers of pupils from grades 1–9 in comprehensive schools. Table 1 gives the background information about the participants, including gender, overall attitude to DC, and the frequency of communicating in that way. In general, parents and teachers were positive toward DC, although the teachers would have liked more frequent contact than the parents. 14% of the teachers answered that “DC increases my workload” and 12% responded “DC is difficult because of misinterprepatations with parents.”

**TABLE 1:** Participants, attitudes to DC, and wanted frequency of contacts via DC.

	**Parents *N* = 400 *n* (%)**	**Teachers *N* = 80 *n* (%)**
**Gender**		
Female	315 (79)	65 (81)
Male	85 (21)	15 (19)
**Attitude to DC**		
Positive/Neutral	330 (82)	62 (78)
Negative	70 (18)	18 (22)
**Wanted frequency of contact via DC**		
Weekly	170 (43)	56 (70)
Monthly	230 (57)	24 (30)

Both quantitative measures and qualitative, open-ended questions were included in the questionnaire to provide a thorough picture of the parents’ and the teachers’ experiences. Below we analyze and discuss the qualitative findings: the quantitative questionnaire and the main quantitative outcomes are reported elsewhere (Kuusimäki et al., 2019). In order to get the widest range of answers, we asked three open questions from parents and teachers. Specifically, parents and teachers were asked to respond to the following questions: (1) What kind of matters would you like to be communicated digitally? (2) What kind of matters would you not like to be communicated digitally? (3) What else would you like to say about the nature of DC?

### Analysis

We subjected the qualitative data to content analysis, the purpose of which is to make replicable and valid inferences from a text (Krippendorf, 2004). The unit of analysis may vary from words to entire interviews (Elo and Kyngäs, 2008). The analysis unit for this study was the aggregate statements that parents and teachers gave to the open-ended questions about their views and expectations to content of DC. The length of responses varied from a few words to several sentences. The analysis was carried out in an inductive-oriented manner as all the codes were derived from the data (Elo and Kyngäs, 2008). The purpose was not to test any theory, but rather to reveal different conceptions of DC that parents and teachers expressed in their own words.

The analysis proceeded in three phases. First, we reduced all the aggregate statements to codes (see Examples 1 and 2). The codes were based on the data, and every time a new topic emerged a new code was created. This coding phase included multiple readings of the data. Statements that included many kinds of codes were divided among different categories accordingly.

Example 1: “If a child is being late, forgetting school materials or disturbing constantly, parents have to be informed.” (aggregate statement from a parent)

• being late, forgetting, disturbing constantly (code)
⇒ Continuous misbehavior (subcategory)

Example 2: “If pupils forget occasionally to do homework or they arrive a bit late to lesson (and these are not symptoms of something more serious), it’s not worth sending parents a message.” (aggregate statement from a teacher)

• forget occasionally, arrive a bit late, it’s not worth sending parents a message (code)
⇒ Infrequent misbehavior (subcategory)

The subcategories thus formed were further combined in three main categories (Table 2). The main categories were named according to contents of subcategories (Elo and Kyngäs, 2008), in these example cases (1) and (2), the main category was named Behavioral issues. The subcategories were formed when possible. Responses concerning quality of feedback formed only three main categories (Table 3). The first author coded the data. The parental data was categorized first, and this categorization guided the analysis of the teacher data. To increase the reliability of the categorizations the second author independently rated 10% of the parental data, and the third author independently rated 50% of the teachers’ data. Interrater reliability was confirmed by means of Cohen’s Kappa (0.71–1.0), which was calculated separately for each category (Cohen, 1960).

**TABLE 2:** The views and expectations of parents and teachers concerning the contents of DC.

	**Number of parents referring to the category (*N* = 400) *n***	**Number of teachers referring to the category (*N* = 80) *n***
**(1) Study-related matters**		
(1.1) Homework, test dates, evaluation, absences	43	16
(1.2) All issues affecting the child’s studies that require parental support	72	22
(1.3) Information about class/school events	27	8
**(2) Behavioral issues**		
(2.1) Continuous misbehavior	16	4
(2.2) Infrequent misbehavior	42	12
**(3) Sensitive issues**		
(3.1) Conflicts	31	11
(3.2) Health issues	20	8

**TABLE 3:** Parents’ and teachers’ expectations of how pupil feedback should be expressed in DC.

	**Number of parents referring to the category (*N* = 400) *n***	**Number of teachers referring to the category (*N* = 80) *n***
(1) Good balance between positive and corrective feedback	22	7
(2) More positive feedback	69	9
(3) Constant emphasis on the child’s weaknesses	32	

## Results

### The Views and Expectations of Parents and Teachers Concerning the Contents of DC

The content analysis revealed three main categories (see Table 2). The biggest one (*n* = 188) concerned *Study-related matters* in both groups, including issues such as homework, test dates, evaluation, and absences. The following examples reflect the definitions of the codes that were classified under this category: “Information about homework, the timetable and the contents of tests” (Parent) and “For example, information about goals, aims, and evaluation” (Teacher). Some teachers saw the digital platform as an evaluation tool, as this example points out: “The digital platform should be developed to archive evaluation about project works, group work, and pupils’ attendance at lessons. It could give continuous information to parents about pupils’ development in learning during the semester.” The category also included various issues related to learning that require cooperation from parents and teachers and parental support. These statements refer to this category: “I need information about how to support my child in his studies, in what subjects and in what way. I would also like to know what they are studying every week, so I could discuss the contents with my child” (Parent) and “The teacher’s weekly digital letter, which contains all kinds of educational and pedagogical issues to be discussed at home” (Teacher). Accordingly, teachers in difficult situations appreciated parents’ cooperation and DC could deliver “information about pupils’ challenges where parents’ support is needed” (Teacher). *Study-related matters* also included information about class/school events. The following examples reflect the definitions used to describe this category: “I need information about my child’s class events and changes in the timetables” (Parent), and “Information about trips, events and timetables” (Teacher).

The second largest category, *Behavioral issues* (*n* = 58), included aspects such as continuous misbehavior and infrequent misbehavior. The following statements exemplify continuous misbehavior: “If being late for lessons, forgetting school materials and misbehavior are becoming frequent, parents have to be informed” (Parent), and “If a child clearly has problems with interaction and in social situations” (Teacher). Informing parents about frequent misbehavior was seen important as this example reveals: “Parents have to be informed about pupils’ ongoing disturbance at lessons or about having continuous conflicts with other children in order to avoid negative surprises concerning child’s behavior” (Teacher). The following statements describe the teachers’ contentment to DC: “The use of DC has considerably decreased misbehavior at lessons, as parents can immediately see teachers’ feedback about the pupil’s behavior.”

The statements in category Infrequent misbehavior covered issues that parents and teachers agreed were minor, that did not need to be communicated digitally. Infrequent misbehavior is described as follows: “I don’t need information about minor behavioral matters such as ‘he went outside without a jacket’… some teachers are in the wrong profession and DC shows that” (Parent). These following statements exemplify the teachers’ views of informing Infrequent misbehavior: “I don’t send messages about minor things that are part of my educational work with a child” and “To my opinion, teachers don’t have to inform parents every day if pupils forget to do homework or forget their school materials. This burdens teachers’ work too much.”

The third category, *Sensitive issues* (*n* = 51), concerned conflicts and health matters. Parents and teachers agreed that the issues included in this category should be dealt with by phone or in face-to-face discussions, and not digitally. The following examples describe conflicts: “In bullying situations or conflicts we want to be contacted by phone immediately” (Parent), and “Discussions about conflicts by phone or inviting the parents to the school, because written communication can lead to misunderstandings” (Teacher). The following examples refer to health issues: “I don’t want to receive messages form a teacher evaluating a child’s personality, or sending sensitive information about their mental health” (Parent), and “Information about confidential matters concerning a pupil’s overall situation in school” (Teacher) or “Pupils personal issues, such as special education plans or psychologist’s statements, have to be informed otherwise than in DC” (Teacher).

### Parental and Teachers’ Expectations of How Pupil Feedback Should Be Expressed in DC

It is not only the contents of DC that affect the parent–teacher partnership but also the nature of the feedback: 78 respondents emphasized the importance of giving positive feedback to children. The following statements illustrate this point: “We would like to have more positive feedback. Receiving encouraging feedback is really important for our youngsters” (Parent), and “Digital platforms should be developed to give positive feedback faster and more easily” (Teacher), or “DC could be more positive. Encouraging feedback uplifts pupils. Constant negative feedback strengthens the negative image about the child.” (Teacher)

This issue was also present in the parents’ responses concerning a constant emphasis on child’s weaknesses (*n* = 32), as these parent’s statements exemplifies: “At this moment we only get negative feedback. Only one teacher sometimes sends positive messages, and it has encouraged our child to become more actively involved,” and “My child is constantly criticized by certain teachers because no one controls DC. Some teachers see only problems.”

Both parents and teachers (*n* = 29) also emphasized the importance of maintaining a good balance in digital feedback, and the following statements illustrate the definitions of balanced feedback: “I find that DC works great! It is important to get both positive and ‘negative’ feedback and I hope I will get information in good time about the things that need to be developed (in the child’s behavior or studies)” (Parent), and “It is important to give realistic and not just positive feedback, otherwise parents may have too rosy an image of the child’s studying and behavior” (Teacher), or “Digital feedback have to be in balance, focusing only on negative or positive gives parents wrong information about the child’s progress. I think DC is a good supplementary tool for communication, but meetings and phone calls are more important.”(Teacher)

## Discussion

This article presents the results of an analysis of the views and expectations of Finnish parents and teachers concerning the content of digital messages and the nature of feedback in DC. We found three main areas of its relevance or non-relevance. It seems that most issues concerning school-related matters or behavioral issues can be appropriately dealt digitally. The results are in line with our previous study, which found that parents and teachers are overall satisfied with DC and that it serves their partnership well by providing versatile information about pupils’ studies and happenings at school (Kuusimäki et al., 2019). Effective and frequent two-way communication supports positive partnership among parents and teachers by conveying information about what is going on at school and at home (Epstein et al., 2009; Chu, 2018). It also allows them to intervene in pupils’ learning and behavioral problems at an early stage (Carr et al., 2015). However, as evidenced in the present study, teachers should be careful when reporting problems in a pupil’s behavior. The parents seemed unwilling to get information about minor and infrequent misbehavior. On the other hand, parents appreciate having frequent and versatile information about their child’s studies (Kuusimäki et al., 2019). Overall, parents and teachers working together in order to share the responsibilities of a child’s learning and growth can greatly decrease teacher workload (Epstein et al., 2009). The constant and immediate support from parents can affect positively on teacher’s everyday work and well-being.

Parents want to have information about their child’s conflicts at school (Kuusimäki et al., 2019). However, according to the present study, both parents and teachers felt that sensitive issues with pupils, like constant conflicts and health issues, should be communicated face-to-face or by phone. Choosing the right channel to communicate is essential because parents and teachers should have open and proactive communication about sensitive issues affecting children’s well-being (Juniu, 2009). It is very important that teachers recognize the conflicts and sensitive matters that are best dealt with by phone or in face-to-face meetings (Palts and Kalmus, 2015). Communicating about sensitive issues concerning a pupil’s conflicts or health issues can cause misunderstandings between parents and teachers. This can be a major issue affecting the partnership and teacher’s well-being because experiences of not being trusted or being criticized by parents may cause anxiety among teachers (Skaalvik and Skaalvik, 2011). Given the large number of sensitive matters that require attention in this study, it would seem that DC alone does not suffice to foster the parent–teacher partnership, and that teachers still need to meet parents personally. The growing demands connected to using the appropriate communication channel for various kinds of information can directly affect the teacher’s workload and well-being.

In this study, parents and teachers expressed the need for more balanced and encouraging feedback on pupils. It appears from our results that there is too much emphasis still on a child’s weaknesses. This result confirms the outcomes from our previous study about parents wanting more encouraging feedback about their children (Kuusimäki et al., 2019). The Finnish study by Oinas et al. (2017) showed that the distribution of feedback is uneven and the majority of pupils received only occasional encouragement. Parents need supportive and encouraging feedback about their child’s studies and development (NBE, 2014). More positive feedback and encouragement might increase well-being in school communities and promote teacher well-being by building partnership and strengthening social relationships with parents.

The specific aim of the present study was to find out what kind of contents parents and teachers wish to communicate digitally and how pupil feedback should be expressed. The results were studied with regard to teacher well-being. To conclude, respectful and trusty DC that also supports teacher well-being contains the elements of frequency, clarity, prudence, proactivity, and encouraging feedback. These findings are in line with the previous studies of parent–teacher communication (Epstein et al., 2009; Juniu, 2009). We claim that DC can be one positive factor building parent–teacher partnership and enhancing teacher well-being. In order to build up fluent DC, there is a need of more studied information of the expectations of parents and teachers on the content and of frequency of DC. By raising teachers’ awareness of parents’ views, teachers can enhance their DC competences. Finnish teacher education lacks training in communication competences and in usage of DC (Alanko, 2018). It is obvious that DC needs to be addressed more carefully in future teacher education. “Best practices” in DC ought to be taught for student teachers as well as for teachers in the field (Epstein, 2018). This is not only to avoid difficulties but to promote partnerships, mutual support and well-being. According to studies, adequate teaching competences in using information and communication technologies (ICT) is factor in teacher well-being (De Pablos-Pons et al., 2012). With good communication skills, it is possible to enhance positive partnership, mutual respect, and trust among teachers and all families (Epstein, 2013). Yet communication skills are not enough; schools need to reflect on common policies in DC and in what time teachers conduct communication with parents, so it does not become another burden on the teacher well-being.

### Limitations

This study provides new, qualitative information on the nature of DC between Finnish parents and teachers. The findings add more detailed information to that reported in our previous study on general trends in DC in Finnish schools (Kuusimäki et al., 2019). We believe the results are reliable, and that they contribute to the discussion on digital home-school communication. However, some limitations should be mentioned. First, it seems that those who chose to participate in this study have a predominantly positive attitude toward DC, which may have biased the responses underlining its advantages. In order to generalize our results, having more municipalities participating in the study would give a more comprehensive picture. In the future, studies on parent–teacher communication need to include more variety in locations and school contexts. Second, there was much less data on teachers than on parents, which may reflect the heavy workload of teachers. The study was executed in May, which is the last month before summer holiday in Finland. In the future, by implementing the study earlier in semester and increasing response time, there could be more responses from teachers. Teachers’ voice could be more in evidence in this study. Third, in some cases the coding was challenging due to the richness of the informants’ statements. However, the three authors coded the data independently, and the kappa values indicated a good inter-rater reliability for the established categories.

Future studies should yield more information about the effect of DC on teacher well-being. Good practices need to be established to develop a healthy work and life balance.

## Data Availability Statement

The data used to support the findings of this study are available on request to the corresponding author.

## Ethics Statement

In our study, all national regulations on research ethics (Finnish Advisory Board on Research Integrity, 2009) were carefully followed. Ethics approval was not needed, according to our national regulations as well as to the Ethical board of the University of Helsinki.

## Author Contributions

A-MK, LU-M, and KT developed the research project. A-MK and LU-M developed the administration procedure. A-MK carried out the data analysis with the contributions of LU-M and KT.

## Conflict of Interest

The authors declare that the research was conducted in the absence of any commercial or financial relationships that could be construed as a potential conflict of interest.

## References

[B1] Agger B. (2011). iTime: labor and life in a smartphone era. *Time Soc.* 20 119–136. 10.1177/0961463X103380730

[B2] Alanko A. (2018). Preparing pre-service teachers for home-school cooperation: exploring Finnish teachers education programmes. *J. Educ. Teach.* 44 321–332. 10.1080/02607476.2018.1465644

[B3] Bauch J. P. (1998). “Applications of technology to linking schools, families, and students,” in *Proceedings of the Families, Technology, and Education Conference*, Chicago, IL.

[B4] Benevene P. Fiorilli C. (2015). Burnout syndrome at school: a comparison study with lay and consecrated Italian teachers. *Mediterr. J. Soc. Sci.* 6 501–506. 10.5901/mjss.2015.v6n1p501

[B5] Benevene P. Wong Y. H. Fiorilli C. De Stasio S. (2018). A cross-national comparison on subjective well-being of kindergarten teachers: Hong Kong and Italy. *Front. Psychol.* 9:2626. 10.3389/fpsyg.2018.02626 30631298 PMC6315170

[B6] Carr N. Heath D. Maghrabi R. (2015). Implications of information and communication technologies (ICT) for school-home communication. *J. Inform. Technol. Educ. Res.* 14 363–396. 10.2894/2285

[B7] Chang M.-L. (2009). An appraisal perspective of teacher burnout: examining the emotional work of teachers. *Educ. Psychol. Rev.* 21 193–218. 10.1007/s10648-009-9106-y

[B8] Chu S.-Y. (2018). Perspectives from both sides of the parent–professional partnership: a preliminary study on Taiwan’s early childhood special education services. *Int. J. Disabil. Dev. Educ.* 65 355–372. 10.1080/1034912X.2017.1403572

[B9] Cohen J. (1960). A coefficient of agreement for nominal scales. *Educ. Psychol. Measure.* 20 37–46. 10.1177/001316446002000104

[B10] De Pablos-Pons J. Colás-Bravo P. González-Ramírez T. Martínez-Vara del Rey C. C. (2012). Teacher well-being and innovation with information and communication technologies; proposal for a structural model. *Qual. Quant.* 47 2755–2767. 10.1007/s11135-012-9686-3

[B11] Elo S. Kyngäs H. (2008). The qualitative content analysis process. *J. Adv. Nurs.* 62 107–115. 10.1111/j.1365-2648.2007.04569.x 18352969

[B12] Epstein J. L. (1995). School, family and community partnership. *Phi Delta Kappan Bloomington* 76 702–712.

[B13] Epstein J. L. (2011). *School, Family, and Community Partnerships: Preparing Educators and Improving Schools*, 2nd Edn New York, NY: Avalon Publishing.

[B14] Epstein J. L. (2013). Ready or not? Preparing future educators for school, family, and community partnerships. *Teach. Educ.* 24 115–118. 10.1080/10476210.2013.786887

[B15] Epstein J. L. (2018). School, family, and community partnerships in teacher’s professional work. *J. Educ. Teach.* 44 398–406.

[B16] Epstein J. L. Sanders M. G. Simon B. S. Salinas K. C. Rodriguez Jansorn N. Van Voorhis F. L. (2009). *School, Family, and Community Partnerships: Your Handbook For Action*, 3rd Edn Thousand Oaks, CA: Corwin press.

[B17] Finnish Advisory Board on Research Integrity (2009). *Ethical Principles of Research in the Humanities and Social and Behavioural Sciences and Proposals for Ethical Review.* Available at: https://www.tenk.fi/en/ethical-review-in-human-sciences (accessed July 20, 2019).

[B18] Freytag C. E. (2001). “Teacher-parent communication: Starting the year off right,” in *Paper Presented at the Biennial Meeting of the Kappa Delta Pi International Honor Society in Education*, Orlando, FL.

[B19] Fusco P. (2004). Connecticut district revamps web site with finalsite. *Journal* 31:28.

[B20] Hakanen J. Ropponen A. Scaufeli W. B. De Witte H. (2018). Who is engaged at work? A large-scale study in 30 european countries. *J. Occup. Environ. Med.* 61 373–381. 10.1097/JOM.0000000000001528 30557226

[B21] Juniu S. (2009). Computer mediated parent-teacher communication. *Rev. Elec. Actual. Invest. Educ.* 9 1–19. 16813041

[B22] Konu A. Rimpelä M. (2002). Well-being in school: a conceptual model. *Health Promot. Int.* 17 79–85. 11847141 10.1093/heapro/17.1.79

[B23] Krippendorf K. (2004). Reliability in content analysis: some common misconseptions and recommendations. *Hum. Commun. Res.* 30 411–433. 10.1111/j.1468-2958.2004.tb00738.x

[B24] Kuusimäki A.-M. Uusitalo-Malmivaara L. Tirri K. (2019). Parents’ and teachers’ views on digital communication in Finland. *Educ. Res. Int.* 7:8236786 10.1155/2019/8236786

[B25] Kvale S. (1996). *Interview Views: An Introduction to Qualitative Research Interviewing.* Thousand Oaks, CA: Sage Publications.

[B26] Maslach C. Jackson S. E. Leiter M. P. (1996). *Maslach Burnout Inventory Manual*, 3rd Edn Mountain View, CA: CPP, Inc.

[B27] NBE (2014). *National Core Curriculum for Basic Education.* Helsinki: Finnish National Board of Education Available at: http://www.oph.fi/download/163777_perusopetuksen_opetussuunnitelman_perusteet_2014.pdf

[B28] OAJ (2017). *Trade Union of The Education.* Available at: https://www.oaj.fi/globalassets/julkaisut/2018/tyoolobarometri_final_0905_sivut.pdf

[B29] Oinas S. Vainikainen M.-P. Hotulainen R. (2017). Technology-enhanced feedback for pupils and parents in Finnish basic education. *Comp. Educ.* 108 59–70. 10.1016/j.compedu.2017.01.012

[B30] Oostdam R. Hooge E. (2013). Making the difference with active parenting. Forming educational partnerships between parents and schools. *Eur. J. Psychol. Educ.* 28 337–351. 10.1007/s10212-012-0117-6 25917810

[B31] Palts K. Kalmus V. (2015). Digital channels in teacher-parent communication: the case of Estonia. *Int. J. Educ. Dev. Inform. Commun. Technol.* 11 65–81.

[B32] Ramirez F. (2001). Technology and parental involvement. *Fred Clear. House.* 75:30.

[B33] Reddy R. Rhodes J. E. Mulhall P. (2003). The influence of teacher support on student adjustment in the middle school years: a latent growth curve study. *Dev. Psychopathol.* 15 119–138. 10.1017/S0954579403000075 12848438

[B34] Samdal O. (1998). *The School Environment as a Risk or Resource for Students’ Health-Related Behaviours and Subjective Well-being.* Bergen: Research Center for Health Promotion, Faculty of Psychology, University of Bergen.

[B35] Samdal O. Torsheim T. (2012). The school environment as a risk or resource for students’ health- related behaviours and subjective well-being. An ecological perspective on health promotion systems, settings and social processes. *Res. Center Health Promot. Faculty Psychol. Univ. Bergen* 48–59. 10.2174/978160805341411201010048

[B36] Scheopner A. J. (2010). Irreconcilable differences: teacher attrition in public and catholic schools. *Educ. Res. Rev.* 5 261–277. 10.1016/j.edurev.2010.03.001

[B37] Skaalvik E. M. Skaalvik S. (2011). Teacher job satisfaction and motivation to leave the teaching profession: Relations with school context, feeling of belonging, and emotional exhaustion. *Teach. Teach. Educ.* 27 1029–1038. 10.1016/j.tate.2011.04.001

[B38] Stoeber J. Rennert D. (2008). Perfectionism in school teachers: relations with stress appraisal, coping styles, and burnout. *Anxiety Stress Coping* 21 37–53. 10.1080/10615800701742461 18027123

[B39] Tirri K. (2014). The last 40 years in Finnish teacher education. *J. Educ. Teach.* 40 600–609. 10.1080/02607476.2014.956545

[B40] Weinstein P. (2005). All in the family: new technologies, federal legislation, and persistent parents have spurred a vast array of products for school-to-home communication. *Technol. Learn.* 25:7.

